# Development of a novel linear model for predicting recipient’s post-transplant serum creatinine level after living donor kidney transplantation: A multicenter cross-validation study

**DOI:** 10.1371/journal.pone.0214247

**Published:** 2019-04-18

**Authors:** Jinsoo Rhu, Sung Joo Kim, Kyo Won Lee, Jae Berm Park, Kyunga Kim, Heejin Yoo, Hyejin Mo, Chanjoong Choi, Sang-il Min, Jongwon Ha

**Affiliations:** 1 Department of Surgery, Samsung Medical Center, Sungkyunkwan University School of Medicine, Seoul, Korea; 2 Statistics and Data Center, Research Institute for Future Medicine, Samsung Medical Center, Seoul, Korea; 3 Department of Surgery, Seoul National University College of Medicine, Seoul Korea; University of Toledo, UNITED STATES

## Abstract

**Background:**

This study was designed to develop and cross-validate a statistical model for predicting post-transplant serum creatinine of living donor kidney transplantation.

**Materials and methods:**

Adult recipients of living donor kidney transplantation from August 2012 to October 2017 at Samsung Medical Center (SMC) and Seoul National University Hospital (SNUH) with normal post-transplant protocol biopsy were included for modelling. Demographic data including recipient and donor’s sex, age, body measurements and comorbidities, pre-transplant donor serum creatinine, graft weight, post-transplant recipient serum creatinine and the result of protocol biopsy were collected. Multivariate linear regression analysis was performed for developing the model based on SMC cohort. Internal validation was performed using leave-one-out cross-validation with the same cohort. External validation using leave-one-out cross-validation was performed based on the cohort of SNUH.

**Results:**

A total of 238 and 191 recipients were included from SMC and SNUH, respectively. The prediction model included recipient’s sex (β = 0.228, P<0.001), height (β = 0.007, P<0.001), and weight (β = 0.006, P<0.001), donor’s age (β = 0.004, P<0.001), height (β = -0.007, P<0.001), pre-transplant serum Cr (β = 0.377, P<0.001) and graft weight (β = -0.002, P<0.001). The model showed R^2^ of 0.708, root mean square error of prediction (RMSEP) of 0.161 and intraclass correlation coefficient (ICC) of 0.83. The internal validation showed predicted ICC of 0.82, RMSEP of 0.161, and accuracy was calculated 0.895. The external validation showed predicted ICC of 0.78, RMSEP of 0.170, and accuracy was calculated 0.876.

**Conclusions:**

The linear prediction model based on body measurement and donor serum creatinine and graft weight showed a high accuracy in cross-validation.

## Introduction

Given refinements in surgical procedures and the development of delicate immunosuppressive strategies, kidney transplantation (KT) has become the best treatment for patients with chronic kidney disease. It also has low associated morbidity and mortality. Due to a shortage of organs from cadaveric donors, living donor KT has also become an alternative option for these patients. After successful KT, recipients experience changes in body homeostasis, with particular regard to water balance and metabolism. This can objectively be measured based on increased urine output and decreased serum blood urea nitrogen and creatinine (Cr). The Cr level drops and reaches a plateau several days after successful KT. However, the level of serum Cr has certain intra- and interpatient variations and can also be influenced by the functional capacity of the kidney graft.

Post-transplant serum Cr is dependent on many surrounding factors, including both recipient-dependent and donor-dependent factors. The previously reported recipient-dependent factors regarding graft function are recipient sex[1–3], body weight[4], height, body surface area[5,6], lean body weight, and body mass index (BMI).[7,8] These factors are representative of the recipient’s metabolic demand. Previously reported donor-dependent factors regarding graft function are donor sex[1,2,9], age, body weight, and graft weight.[6,10] These factors represent the metabolic demand of the donor.

**Fig 1:**
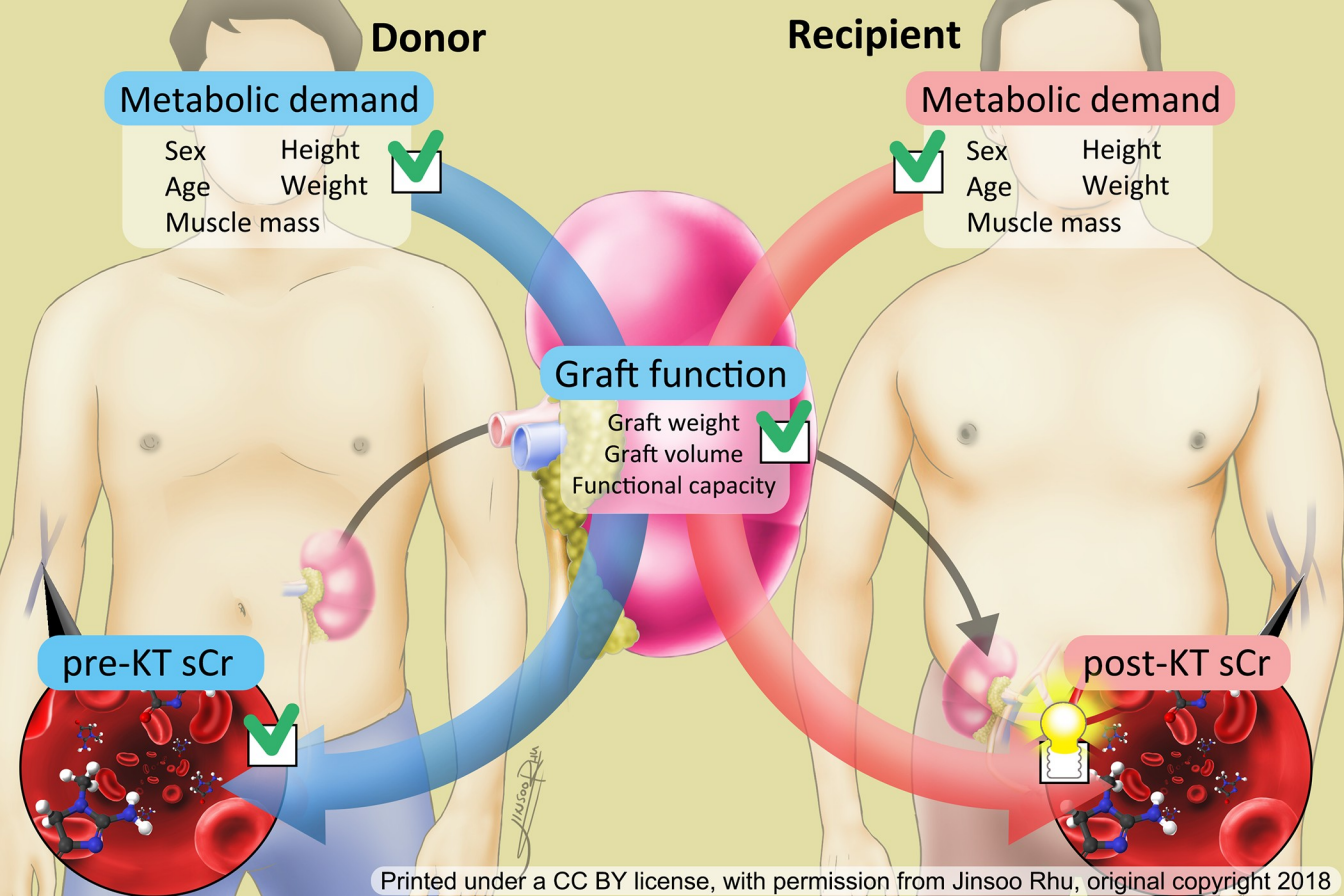
Brief concept of the study hypothesis. The function of the graft kidney includes the graft weight, which represents the nephron mass, and its functional capacity. The graft weight can be measured during transplantation. The volume is calculated using computed tomography. The functional capacity of the graft kidney can be estimated with regard to the metabolic demand of the donor and donor’s pre-transplant serum creatinine. Therefore, the post-transplant serum creatinine level of the recipient can be estimated by replacing the data from the donor’s metabolic demand with that of the recipient. Green check points represent data that can be measured. The post-transplant serum creatinine of the recipient is calculated based on pre-existing data. **KT** kidney transplantation, **sCr** serum creatinine.

Oh et al. published a study in 2005 that showed a statistical relationship between the factors representative of metabolic demand and renal mass supply to early graft function.[6] Although this model demonstrated promising results, it was not internally or externally validated. The recipient’s post-KT serum Cr reflects the functional outcome of the graft kidney’s performance by filtering the metabolic demand of the recipient. Therefore, we hypothesized that the graft kidney’s performance could be estimated based on the donor’s serum Cr prior to KT, the metabolic demand of the donor, and the graft weight (which reflects the nephron mass). If these hypotheses are correct, one could build a model to predict the recipient’s post-KT serum Cr based on the physical data of both donor and recipients using the renal graft weight and donor serum Cr. (Fig 1) Therefore, we designed this retrospective multicenter study to build and cross-validate a statistical model for predicting the post-KT serum Cr of living donor KT.

## Materials and methods

This study was approved by the Institutional Review Boards of SMC (IRB No. 2018-03-054) and SNUH (IRB No. 1805-126-948). The need for informed consent was waived by the both institutional review boards since the study was designed as a retrospective study. None of the transplant donors were from a vulnerable population and all donors provided written informed consent that was freely given.

### Patients

Patients >18 years of age who underwent living donor KT between August 2012 and October 2017 at Samsung Medical Center (SMC) or Seoul National University Hospital (SNUH) were reviewed. Those recipients who underwent protocol biopsy during their first admission period were reviewed for study inclusion. We only included recipients with normal histology on the protocol biopsy for prediction modelling. Therefore, patients with abnormal pathology, including acute T cell mediated rejection, acute antibody mediated rejection, calcineurin inhibitor (CNI) toxicity, or other abnormal findings that can compromise renal function, were excluded.

### Immunosuppression and protocol biopsy

Although the immunosuppression protocol was similar between SMC and SNUH, there were slight differences. While recipients at SMC mostly underwent induction therapy using antithymocyte globulin, basiliximab was preferred at SNUH. Tacrolimus, mycophenolate mofetil, and methylprednisolone were the main immunosuppressants used in the triple maintenance regimen at both centers.

The protocol biopsy was performed within the second week after KT at both centers. At SMC, the biopsy was performed around the 12th post-KT day. At SNUH, the biopsy was performed around the 9th post-KT day. Recipients with significant bleeding risk but without suspicious signs of comorbidity did not undergo protocol biopsy for safety reasons. At both hospitals, the histopathology was reviewed by expert renal transplant pathologists.

### Data collection

Demographic data including sex, age, height, weight, BMI, comorbidities were collected for recipients and their donors. Cause of renal disease, human leukocyte antigen (HLA) mismatch between donor and recipient, presence of donor specific antibody, donor serum Cr prior to KT, donor kidney graft weight, performance and reason of plasmapheresis, induction therapy, maintenance immunosuppressive regimen, and the result of protocol biopsy were reviewed. Graft weight was measured after completing the bench surgery. Post-KT serum Cr was measured at the day of the protocol biopsy and was log-transformed prior to analysis. Clinical courses of recipients were reviewed based on medical charts and whether they undergo certain complications. The data of the cohort was collected by the data managers of each center, separately.

### Data analysis

Baseline characteristics were summarized via frequencies and percent for categorical variables and mean and standard deviation for continuous variables. Comparison between the two centers (SMC vs SNUH) was performed using chi-square test, Fisher exact test or linear-by-linear association for categorical variables and student’s t-test or Mann-Whitney test for continuous variables as appropriate.

The SMC data was used for model building in predicting post-KT serum Cr based on univariate and multivariate linear regression. Univariate linear regression analysis was performed for each variable. Then the variables with a P<0.10 in the univariate analysis were included in the multivariate analysis. The stepwise selection method was accompanied to identify a best prediction model. The performance of the identified model was assessed by adjusted R^2^ and intraclass correlation coefficient (ICC) between observed and estimated values. Leave-one-out cross-validation was employed for internal validation. We also performed external validation using the SNUH data. For validation, Bland-Altman plot was used along with three predictability measures: the predicted ICC, root mean square error of prediction (RMSEP) and accuracy.[11] The predicted ICC was ICC between observed and predicted values of post-KT serum Cr. Accuracy was calculated as “1 − (RMSEP/range of log-transformed post-KT serum CR)”.

Statistical analyses were performed using SPSS 20.0 (SPSS Inc., Chicago, IL, USA) and SAS v9.4 (SAS Institute Inc, Cary, NC, USA) by expert statisticians of biomedicine in SMC.

## Results

**Fig 2:**
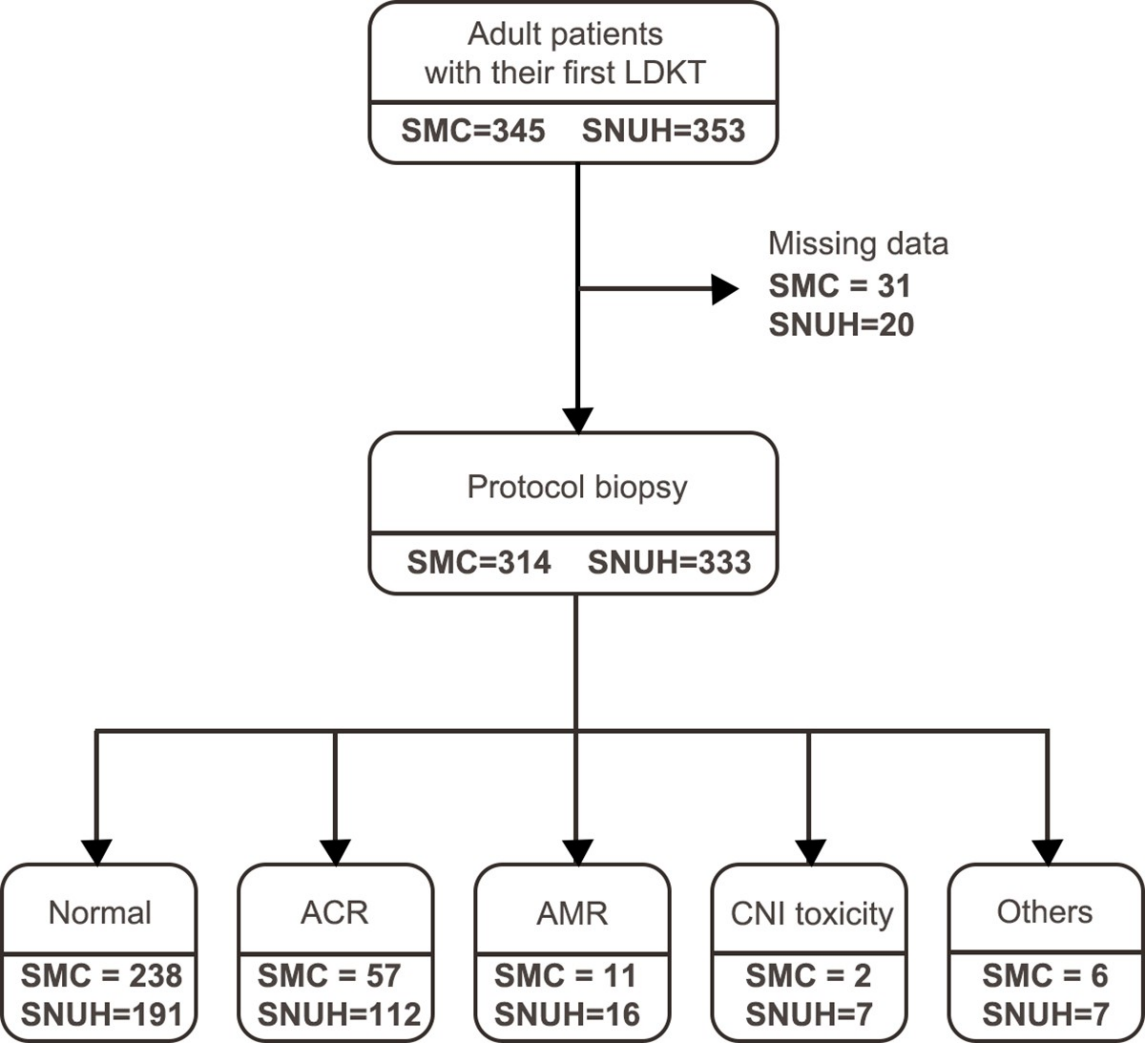
Flow diagram of patient inclusion from Samsung Medical Center and Seoul National University Hospital. **SMC** Samsung Medical Center, **SNUH** Seoul National University Hospital, **ACR** Acute cellular rejection, **AMR** Antibody-mediated rejection, **CNI** Calcineurin inhibitor.

During the study period, a total of 345 and 353 adult recipients underwent living donor KT at SMC and SNUH, respectively. Among these patients, 314 recipients from SMC and 333 from SNUH underwent protocol biopsy. Only 238 and 191 recipients from SMC and SNUH, respectively, had normal histopathology on the protocol biopsy. A total of 76 and 142 recipients from SMC and SNUH, respectively, were excluded from modelling due to abnormal histopathology on protocol biopsy. (Fig 2)

### Comparing recipients from the two centers

Table 1 shows the baseline characteristics from each hospital. There were no statistically significant differences between the hospitals with regard to recipient’s sex, age, height, weight, BMI, diabetes, or other underlying medical problems. However, the rate of polycystic kidney disease was higher in the recipients at SNUH (n = 21, 11.0%) than it was in those from SMC (n = 9, 2.8%, P = 0.004). There was no significant difference between the two groups with regard to the donor-recipient data, including HLA mismatches, panel reactive antibody, and donor specific antibody. There were no significant differences in the donor characteristics, including donor serum Cr, prior to KT and kidney graft weight.

**Table 1:** Comparisons of baseline characteristics and clinical courses of the patient groups.

Factors	SMC (n = 238)	SNUH (n = 191)	P-value
Recipient			
Sex (Male/Female)	130/108	120/71	0.087
Age (years), mean ± SD	47.5 ± 12.0	45.6 ± 12.7	0.127
Height (cm), mean ± SD	164.1 ± 9.5	165.7 ± 9.0	0.080
Weight (kg), mean ± SD	62.0 ± 13.3	62.8 ± 12.9	0.508
BMI(kg/m^2^), mean ± SD	22.9 ± 3.7	22.8 ± 3.8	0.832
Diabetes	72 (30.3%)	42 (22.0%)	0.054
Hepatitis B carrier	11 (3.4%)	9 (4.7%)	0.965
Hepatitis C carrier	3 (1.3%)	0 (0.0%)	0.259
History of cardiovascular accident	15 (6.5%)	18 (9.4%)	0.264
History of cerebrovascular accident	6 (2.6%)	4 (2.1%)	0.749
Renal replacement therapy			
Dialysis (Hemodialysis/Peritoneal dialysis)	160 (148/12)	137 (115/22)	
No dialysis	78	54	
Underlying kidney disease			
Diabetic nephropathy	69 (29.0%)	41 (21.5%)	0.076
IgA nephropathy	45 (18.9%)	43 (22.5%)	0.358
Focal segmental glomerulosclerosis	7 (2.9%)	8 (4.2%)	0.485
Other glomerulonephritis	30 (12.6%)	26 (13.6%)	0.758
Polycystic kidney disease	9 (3.8%)	21 (11.0%)	0.004
Hypertensive nephropathy	36 (15.1%)	18 (9.4%)	0.077
Others	9 (2.8%)	10 (5.2%)	0.467
Unknown	33 (13.9%)	24 (12.6%)	0.693
Recipient-Donor relationship			
Parent to child	34 (14.3%)	39 (20.4%)	
Child to parent	56 (23.5%)	25 (13.1%)	
Sibling	54 (22.7%)	61 (31.9%)	
Spouse	72 (30.3%)	45 (23.6%)	
Relatives	22 (9.2%)	21 (11.0%)	
HLA mismatches (mm), n			0.969
0 mm	19 (8.0%)	20 (10.6%)	
1 mm	17 (7.1%)	16 (8.5%)	
2 mm	57 (23.9%)	28 (14.8%)	
3 mm	69 (29.0%)	62 (32.8%)	
4 mm	28 (11.8%)	24 (12.8%)	
5 mm	33 (13.9%)	27 (14.3%)	
6 mm	15 (6.3%)	12 (6.3%)	
HLA mm per patient, mean ± SD	2.96 ± 1.58	2.97 ± 1.65	0.969
Panel reactive antibody			0.260
0%	176 (73.9%)	146 (76.4%)	
1–49%	35 (14.7%)	32 (16.8%)	
≥50%	27 (11.3%)	13 (6.8%)	
Donor specific antibody prior to KT	27 (11.3%)	16 (8.4%)	0.318
Donor			
Sex (M/ F)	117/121	95/96	0.905
Age (years), mean ± SD	43.9 ± 11.8	45.3 ± 11.2	0.175
Height (cm), mean ± SD	164.4 ± 8.7	164.9 ± 8.7	0.598
Weight (kg), mean ± SD	65.9 ± 11.6	66.2 ± 11.0	0.870
BMI(kg/m^2^), mean ± SD	24.3 ± 3.1	24.2 ± 2.8	0.870
Donor serum creatinine (mg/dL), mean ± SD	0.81 ± 0.16	0.81 ± 0.16	0.973
Donor kidney graft weight (g), mean ± SD	172.7 ± 33.9	168.2 ± 31.7	0.155
Plasmapheresis prior to KT	59 (24.8%)	40 (20.9%)	0.347
ABO incompatible	46	27	
High MFI DSA	4	13	
Positive flow cytometry	1	-	
Induction therapy			<0.001
Antithymocyte globulin	82 (34.5%)	5 (2.6%)	
Antithymocyte globulin with rituximab	77 (32.4%)	10 (5.2%)	
Basiliximab	78 (32.8%)	135 (70.7%)	
Basiliximab with rituximab	-	39 (20.4%)	
None	1 (0.4%)	-	
Maintenance regimen (FK506 vs. other)			<0.001
FK506/MMF/Steroid	235 (98.7%)	170 (89.5%)	
Cyclosporine/MMF/Steroid	2 (0.8%)	10 (5.3%)	
Other	1 (0.4)	10 (5.3%)	
Mean post-KT day for protocol biopsy	12.6 ± 1.8	9.6 ± 1.6	<0.001
Post-KT serum creatinine (mg/dL), mean	1.02 ± 0.30	0.99 ± 0.27	0.357
Minimum-maximum	0.47–2.20	0.48–1.89	
<1.00	125	100	0.576
1.00–1.49	98	84	
1.50–1.99	13	7	
≥2.00	2	-	

**SMC** Samsung Medical Center, **SNUH** Seoul National University Hospital, **SD** Standard deviation, **BMI** Body mass index, **HLA** Human leukocyte antigen, **KT** Kidney transplantation, **MFI** Mean fluorescence intensity, **DSA** Donor specific antibody, **MMF** Mycophenolate mofetil

However, there were significant differences between the two groups with regard to immunosuppression. While SMC mainly used an antithymocyte globulin-based induction regimen (n = 157, 66.9%), SNUH used a basiliximab-based induction regimen (n = 174, 91.1%, P<0.001). After transplant, 98.7% (n = 235) of recipients at SMC received a tacrolimus-based maintenance regimen, while only 89.5% (n = 170) of those at SNUH were under tacrolimus. (P<0.001) Instead of tacrolimus, 10 recipients at SNUH received a cyclosporine-based maintenance regimen, while the other 10 received a sirolimus-based regimen.

**Fig 3:**
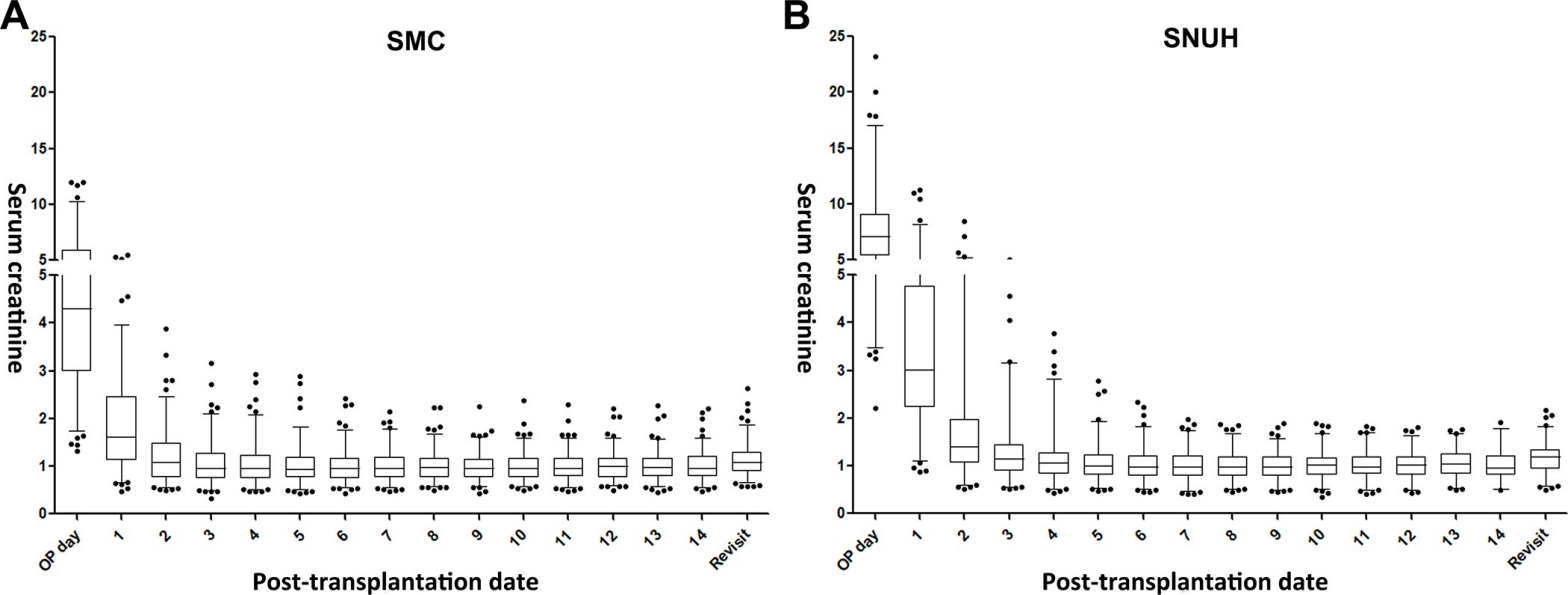
Box and whisker plots of mean post-transplant serum creatinine levels from each group. **SMC** Samsung Medical Center, **SNUH** Seoul National University Hospital, **OP** Operation.

The mean post-KT day for protocol biopsy differed between the two study cohorts (12.6 ± 1.8 days in SMC vs. 9.6 ± 1.6 days in SNUH, P<0.001). However, there was no difference in mean post-KT serum Cr level between SMC (1.02 ± 0.30 mg/dL) and SNUH (0.99 ± 0.27 mg/dL, P = 0.357). Fig 3 shows the mean (±1.96 standard deviations) values of pre- and post-KT serum Cr levels from each center, as well as the serum Cr at the first outpatient clinic visit.

### Prediction modelling based on the SMC cohort

We used univariate linear regression analysis to predict the post-KT serum Cr on the day of protocol biopsy based on variables reflecting the metabolic demand of both the donor and recipient and potential factors that might influence the post-KT graft function. (Table 2) In the univariate analyses, the following parameters were statistically significant: recipient sex (β = 0.409, P<0.001), height (β = 0.020, P<0.001), weight (β = 0.014, P<0.001) and DM (β = 0.109, P = 0.007) and donor sex (β = -0.159, P<0.001), age (β = 0.005, P = 0.004), height (β = -0.009, P<0.001), weight (β = -0.006, P<0.001), graft weight (β = -0.002, P = 0.001), and presence of donor specific antibody (β = -0.145, P = 0.014). Variables with P<0.100 including these variables were included in the multivariate analysis using stepwise method.

**Table 2:** Multivariate linear regression model for predicting the log-transformed post-transplant serum creatinine of the recipient based on potential factors related to graft function.

Factors	Univariate							Final model of multivariate analysis*			
	β	Standard error	R^2^	Adjusted R^2^	RMSEP	Predicted R^2^	P	β	Standard error	P	VIF
Recipient											
Sex (Male vs. female)	0.409	0.027	0.501	0.499	0.205	0.492	<0.001	0.228	0.030	<0.001	2.194
Age	0.000	0.002	0.000	-0.004	0.290	-0.017	0.895				
Height	0.020	0.002	0.412	0.409	0.222	0.402	<0.001	0.007	0.002	<0.001	2.478
Weight	0.014	0.001	0.403	0.401	0.224	0.393	<0.001	0.006	0.001	<0.001	1.955
DM	0.109	0.040	0.031	0.026	0.285	0.015	0.007				
Cardiovascular disease	0.072	0.076	0.004	0.000	0.286	-0.011	0.344				
Cerebrovascular disease	0.025	0.118	0.000	-0.002	0.287	-0.017	0.830				
Donor											
Sex (Male vs. female)	-0.159	0.036	0.077	0.073	0.279	0.061	<0.001				
Age	0.005	0.002	0.035	0.031	0.285	0.019	0.004	0.004	0.001	<0.001	1.490
Height	-0.009	0.002	0.080	0.076	0.278	0.065	<0.001	-0.007	0.002	<0.001	2.774
Weight	-0.006	0.002	0.059	0.055	0.281	0.043	<0.001				
DM	-0.556	0.287	0.016	0.011	0.286	0.007	0.054				
Serum creatinine	-0.193	0.115	0.012	0.008	0.288	-0.005	0.090	0.377	0.086	<0.001	1.835
Graft weight	-0.002	0.001	0.044	0.040	0.284	0.026	0.001	-0.002	0.000	<0.001	1.464
Plasmapheresis	0.027	0.043	0.002	-0.003	0.290	-0.016	0.534				
HLA 1 mismatch	0.028	0.017	0.011	0.007	0.288	-0.004	0.104				
HLA 2 mismatch	0.039	0.026	0.009	0.005	0.289	-0.007	0.139				
Donor specific antibody	-0.145	0.058	0.026	0.021	0.287	0.009	0.014				

**DM** Diabetes mellitus, **HLA** Human leukocyte antigen, **RMSEP** Root mean square error of prediction

In the final model, we included the following parameters: recipient sex (β = 0.228, P<0.001), height (β = 0.007, P<0.001), and weight (β = 0.006, P<0.001) and donor age (β = 0.004, P<0.001), height (β = -0.007, P<0.001), donor serum Cr (β = 0.377, P<0.001), and graft weight (β = -0.002, P<0.001). In contrast, recipient DM, donor sex, and weight and the presence of donor specific antibody were excluded. The final multivariate model had an R^2^ of 0.708, RMSEP of 0.161, and ICC of 0.83 (95%CI 0.79–0.87). The final formula of the prediction model is as follows:

lnCr=0.228×rSex+0.007×rHeight+0.006×rWeight+0.004×dAge−0.007×dHeight+0.377×dCr−0.002×graftweight−0.804

or

$$\mathrm{Cr}=e^{0.228\times rSex+0.007\times rHeight+0.006\times rWeight+0.004\times dAge-0.007\times dHeight+0.377\times dCr-0.002\times graft\;weight-0.804}$$

### Internal validation of the prediction model based on the SMC cohort

**Fig 4:**
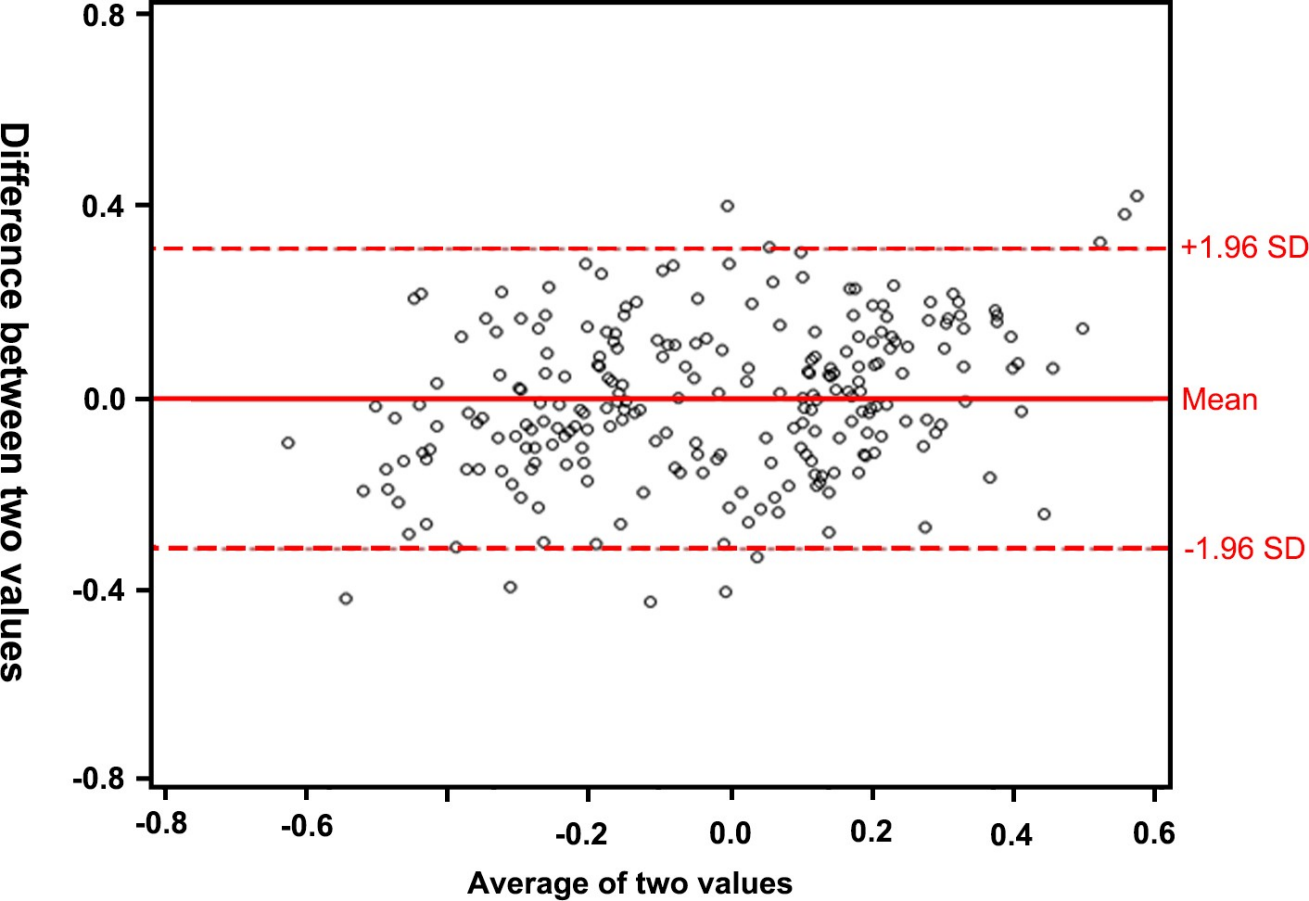
Internal validation of the multivariate linear model based on the cohort from Samsung Medical Center demonstrated using a Bland-Altman plot. Only 10 patients (4.2%) fell outside of the 95% confidence interval of the prediction.

We performed internal validation using leave-one-out cross-validation in the SMC cohort. The predicted ICC was 0.82 (95%CI 0.77–0.85). The RMSEP was 0.161, and the calculated accuracy was 0.895. The Bland-Altman plot demonstrated that only 4.2% (n = 10) of the patients were outside of the mean ± 1.96 standard deviations. (Fig 4)

### External validation of the prediction model based on the SNUH cohort

**Fig 5:**
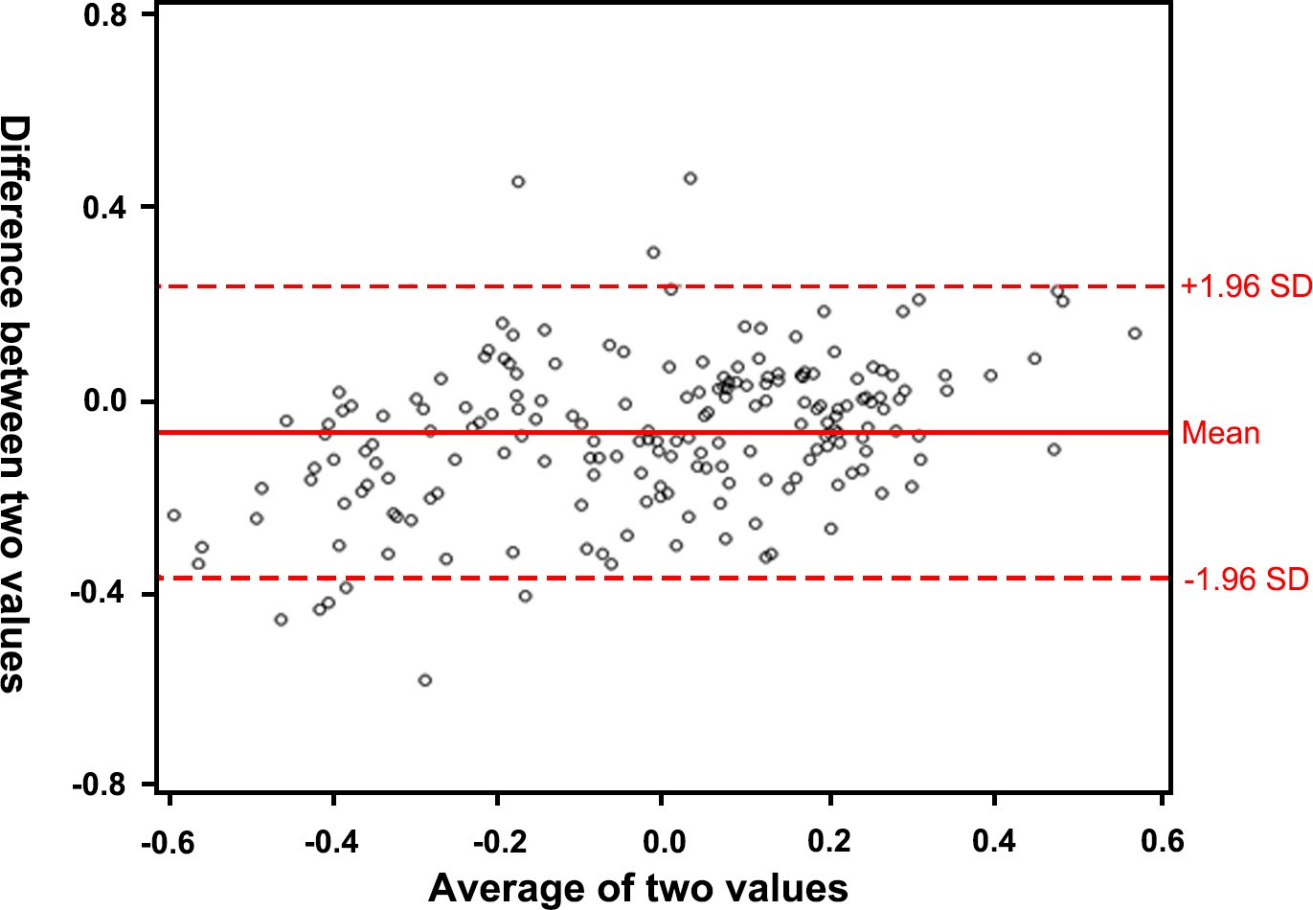
External validation of the multivariate linear model based on the cohort from Seoul National University Hospital demonstrated using a Bland-Altman plot. Only 9 patients (4.7%) fell outside of the 95% confidence interval of the prediction.

**Fig 6:**
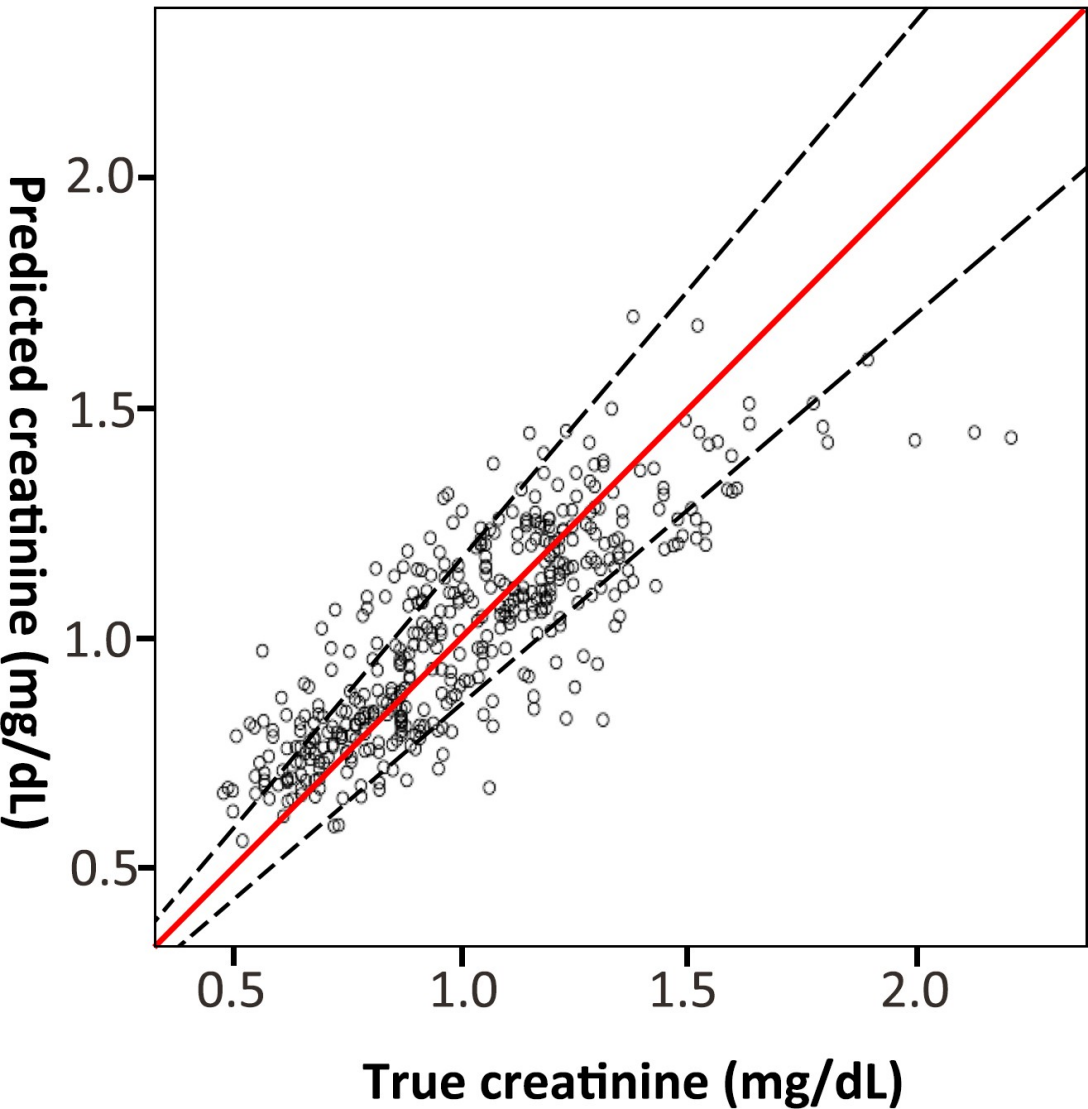
Relationship between the predicted serum creatinine and the true serum creatinine of the study subjects at the time of protocol biopsy.

With regard to the SNUH cohort, we performed external validation using leave-one-out cross-validation. The predicted ICC was 0.78 (95%CI 0.72–0.83). The RMSEP was 0.170, with a calculated accuracy of 0.876. The Bland-Altman plot shows that only 4.7% (n = 9) of the patients were outside of the mean ± 1.96 standard deviations. (Fig 5) Fig 6 shows relationship between the predicted serum creatinine and the true serum creatinine of subject patients.

## Discussion

Since the first successful KT, the transplantation community has overcome numerous hurdles to minimize the morbidity and mortality of KT recipients. Compared to recipients of other solid organ transplants, kidney transplant recipients face additional, complex immunological risks. Therefore, some clinicians, including those at our hospital, have started monitoring the kidney under the microscope by performing protocol biopsy in the early post-KT period. Without performing a biopsy, the only other monitoring method of graft function is the volume of urine produced and serum chemistries (especially serum Cr). However, despite relevant research, there are no well-validated prediction models for recipient post-KT serum Cr. Such a model would be valuable, as it would act as a good index of graft performance.

Therefore, this study was designed to build a prediction model for use (by renal transplant clinicians) immediate post-KT. We were able to exclude cases with complications using the protocol biopsy program during the post-KT admission period. Our model was only designed to predict the post-KT serum Cr level when the graft is under no harm from immunological risk. We hypothesized that we could estimate the post-KT serum Cr if we knew the metabolic demand of the recipient and the graft kidney’s potential performance. This performance can be estimated based on the metabolic demand of the donor, the graft weight of the kidney (which represents the nephron mass), and the donor’s serum Cr (which reflects the functional outcome of graft performance). (Fig 1) Therefore, we analyzed a multivariate model including parameters that are representative of the donor and recipient metabolic demand, along with variables that might influence the graft kidney function after KT. Interestingly, the variables that we predicted would have an influence on the serum Cr were excluded from the final model.

The performance of our prediction model was validated both internally and externally and was found to have high accuracy (of 0.895 and 0.876, respectively). Based on the Bland-Altman plot, <5% of the cohort fell outside of the predicted mean ± 1.96 standard deviation, which is still within the 95% confidence interval. The model had an R^2^ of 0.708 in the initial model, which was higher than that of the previous model described by Oh et al.[6] With the linear model presented in the text, clinicians can easily calculate the predicted serum Cr level for the recipient with the information of recipient’s sex, height, weight, donor’s age, height, pre-transplant serum creatinine, and graft weight. With the information on the graft volume that can be calculated with computed tomography even before transplantation, it is possible to calculate the post-KT serum Cr even before the operation. Although using a graft volume instead of graft weight needs more study, our preliminary analysis including graft volume instead of graft weight showed similar outcome. (S1 Table, S1 and S2 Figs) Due to the lack of data from SNUH, we decided to develop a model using graft weight.

Despite our model’s accuracy and well-validated results, our study still has several limitations. The prediction model was designed based on adult post-KT recipient cohorts from two centers in the Republic of Korea. The mean height, weight, and BMI from SMC and SNUH were 164.1 ± 9.5 cm, 62.0 ± 13.3 kg and 22.9 ± 3.7 kg/m^2^ and 165.7 ± 9.0 cm, 62.8 ± 12.9 kg and 22.8 ± 3.8 kg/m^2^, respectively. Most of the included patients had relatively lean body habitus. Therefore, our model is generalizable to a similar population, but not necessarily to one of larger patients. Therefore, there is a need for separate models for distinct populations (using the same variables), which reflect ethnic characteristics that may influence graft function.

Another limitation of this study is that we did not analyze the model’s discriminating power. Using the protocol biopsy in the early period of post-KT, we found that some patients had abnormal pathology despite normal serum Cr level. The practicality of this model must be validated by analyzing its ability to discriminate normal patients from those with ongoing disease. However, this study was designed to build and validate a prediction model based on patients without events. Therefore, our next research goal is to use the model to address patients with events from those who are uneventful.

The serum Cr level can be influenced by many surrounding factors, especially water homeostasis. However, our prediction model does not address factors that might influence creatinine on a daily basis. We believe that these factors should be adjusted for in further studies. In doing so, the prediction model will be even more clinically useful. Future studies that calculate the donor’s true muscle volume (using computed tomography) may also be interesting.

Although the SMC and SNUH cohorts had small differences, the accuracy values of the models were similar. This accuracy can be explained by our inclusion of patients with normal grafts with similar post-KT serum Cr levels. Despite differing dates of protocol biopsy between the cohorts, (12.6 days in SMC vs. 9.6 days in SNUH) there was no difference in actual Cr level. (1.02 ± 0.30 mg/dL in SMC vs. 0.99 ± 0.27 mg/dL in SNUH)

Despite its limitations, out study is the first to develop a prediction model for post-KT serum Cr of KT recipients and to validate the model both internally and externally. The advantage of this model is that it is based on simple variables that can be obtained with minimal effort. The model’s predictive power may have been increased by including additional parameters that are representative of daily water homeostasis and true muscle volume, although we considered these unnecessary for practical reasons. We believe that our model serves as a baseline study for further investigations into early post-KT complications. Our model also provides baseline data for investigation of the long-term graft function of KT recipients.

## Supporting information

S1 Fig Scatter plot and linear regression model for estimating kidney weight based on kidney volume calculated based on computed tomography. (DOCX)

S2 Fig Scatter plot and linear regression model for estimating predicted post-KT serum creatinine calculated using model with graft weight based on predicted post-KT serum creatinine calculated using model with graft volume. (DOCX)

S1 Table Multivariate linear regression model for predicting post-KT serum creatinine level based on graft weight and graft volume. (DOCX)

## Abbreviations

KT: Kidney transplantation
Cr: Creatinine
SMC: Samsung Medical Center
SNUH: Seoul National University Hospital
CNI: Calcineurin inhibitor
ICC: Intraclass correlation coefficient
RMSEP: Root mean square error of prediction
VIF: Variance inflation factor
