# Supplementary material for: Development of a novel linear model for predicting recipient’s post-transplant serum creatinine level after living donor kidney transplantation: A multicenter cross-validation study
Source: PLoS One. 2019 Apr 18;14(4):e0214247. doi: 10.1371/journal.pone.0214247 (PMC6472729; doi:10.1371/journal.pone.0214247)
Supplement: S1 Fig — (DOCX) [file pone.0214247.s001.docx]

S1 Fig. Scatter plot and linear regression model for estimating kidney weight based on kidney volume calculated based on computed tomography.


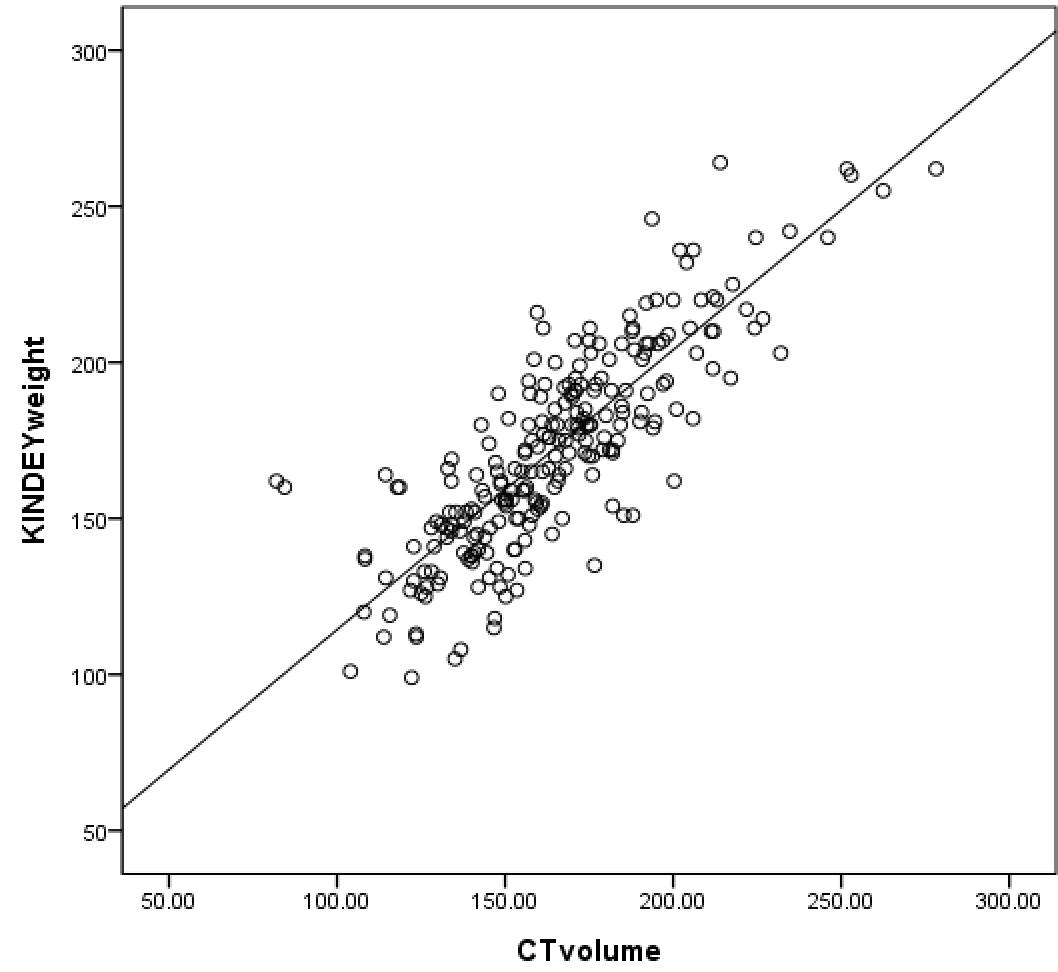


R^2^=0.668, Graft weight=0.897×graft volume+24.737
