# Supplementary material for: Development of a novel linear model for predicting recipient’s post-transplant serum creatinine level after living donor kidney transplantation: A multicenter cross-validation study
Source: PLoS One. 2019 Apr 18;14(4):e0214247. doi: 10.1371/journal.pone.0214247 (PMC6472729; doi:10.1371/journal.pone.0214247)
Supplement: S2 Fig — (DOCX) [file pone.0214247.s002.docx]

S2 Fig. Scatter plot and linear regression model for estimating predicted post-KT serum creatinine calculated using model with graft weight based on predicted post-KT serum creatinine calculated using model with graft volume.


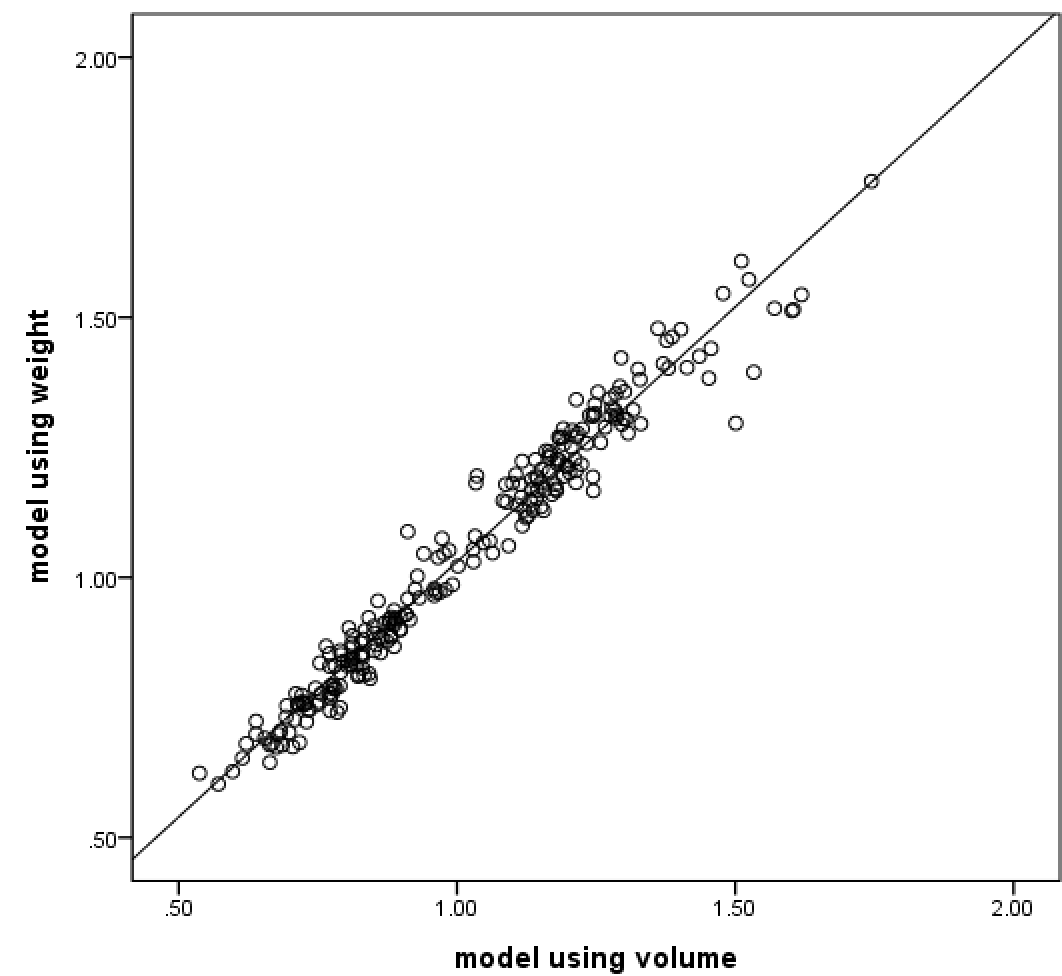


R^2^=0.965,

Estimated creatinine based on model with Graft Weight
= 0.981×(estimated creatinine based on model with graft volume)+0.049
