# Supplementary material for: Development of a novel linear model for predicting recipient’s post-transplant serum creatinine level after living donor kidney transplantation: A multicenter cross-validation study
Source: PLoS One. 2019 Apr 18;14(4):e0214247. doi: 10.1371/journal.pone.0214247 (PMC6472729; doi:10.1371/journal.pone.0214247)
Supplement: S1 Table — (DOCX) [file pone.0214247.s003.docx]

S1 Table. Multivariate linear regression model for predicting post-KT serum creatinine level based on graft weight and graft volume

| Factors | Model with graft weight | | | | Model with graft volume | | | |
| --- | --- | --- | --- | --- | --- | --- | --- | --- |
|  | β | Standard error | P | VIF | β | Standard error | P | VIF |
| Recipient  Sex (Male vs. female)  Height  Weight | 0.228  0.007  0.006 | 0.030  0.002  0.001 | <0.001  <0.001  <0.001 | 2.194  2.478  1.955 | 0.230  0.007  0.006 | 0.031  0.002  0.001 | <0.001  <0.001  <0.001 | 2.195  2.480  1.955 |
| Donor  Age  Height  Serum Creatinine  Graft weight or volume | 0.004  -0.007  0.377  -0.002 | 0.001  0.002  0.086  0.000 | <0.001  <0.001  <0.001  <0.001 | 1.490  2.774  1.835  1.464 | 0.003  -0.006  0.387  -0.002 | 0.001  0.002  0.086  0.000 | 0.003  0.003  <0.001  <0.001 | 1.323  3.008  1.821  1.577 |
| Constant | -0.804 | 0.347 | 0.021 |  | -0.786 | 0.348 | 0.025 |  |
|  | R^2^=0.708 | | | | R^2^=0.706 | | | |

*Donor kidney graft weight (g), mean ± SD =172.7 ± 33.9
 Donor kidney estimated graft volume (cc), mean ± SD =165.0 ± 31.0
